# Provider-Initiated HIV testing and counseling among patients with presumptive tuberculosis in Democratic Republic of Congo

**DOI:** 10.11604/pamj.2016.25.161.8125

**Published:** 2016-11-15

**Authors:** Marcel Yotebieng, Landry Kipula Wenzi, Emmanuel Basaki, Marie Louise Batumbula, Martine Tabala, Eugenie Mungoyo, Richard Mangala, Frieda Behets

**Affiliations:** 1 The Ohio State University, College of Public Health, Division of Epidemiology, Columbus, OH, USA; 2 The University of North Carolina at Chapel Hill, Department of Epidemiology, Chapel Hill, NC, USA; 3 The University of Kinshasa, School of Public Health, Kinshasa, Democratic Republic of Congo; 4 The University of North Carolina at Chapel Hill, School of Medicine, Chapel Hill, NC, USA

**Keywords:** PITC, Presumptive tuberculosis, HIV prevalence, DR Congo

## Abstract

**Introduction:**

Provider-initiated HIV testing and counseling (PITC) of patients with presumptive tuberculosis (TB) is not widely implemented and the burden of HIV among them is not well characterized. We assessed the uptake of PITC and prevalence of HIV among patients with presumptive TB in primary care settings in the Democratic Republic of Congo.

**Methods:**

PITC was implemented in primary care TB clinics in Kinshasa and Kisangani, respectively. In each of the clinics, all patients presenting with cough lasting more than two weeks or any other symptom suggestive of TB were offered HIV testing and counseling and those found to be HIV+ were linked to HIV care and treatment.

**Results:**

Between November 2011 and June 2013, 43,145 patients with presumptive TB were registered in 65 clinics in Kinshasa of whom 84.0% were counseled; 92.4% of those counseled were tested and 4,320 (12.9%) were found to be HIV+. Similarly, in Kisangani, of the 6,687 patients with presumptive TB were registered in 13 clinics, 80.5% were counseled; 99.3% were tested for HIV and 619 (11.6%) were found to be HIV+.

**Conclusion:**

Implementation of PITC among patients with presumptive TB in primary care clinics was associated with high uptake of HIV testing and identification of high number of HIV+ patients.

## Introduction

Tuberculosis (TB) is the main cause of morbidity and mortality among human immunodeficiency virus (HIV) infected persons worldwide [1, 2]. HIV-infected persons are 20-30 times more likely to develop TB disease after becoming infected and one-fourth of all HIV deaths are attributable to TB disease [2]. According to the World Health Organization (WHO), TB accounted for 24% of all deaths among people living with HIV/AIDS (PLWH) in 2013 [3]. Yet, in 2013, only 48% of notified TB patients worldwide had documented HIV test results. To facilitate early and enhanced diagnosis of HIV-infected TB patients, WHO recommends intensified TB case finding at all HIV care settings and provider initiated HIV testing and counseling (PITC) for patients with diagnosed and presumptive TB [4]. Yet, despite the fact that it might help diagnose large numbers of HIV-infected patients who are seeking care for TB like symptoms in TB clinics and potentially speed up TB diagnosis in HIV co-infected patients with smear negative TB [5], PITC for patients with presumptive TB has not been widely implemented, particularly in the high TB and HIV burden countries in sub-Saharan Africa [6]. Consequently, more than a decade after it was first recommended by the WHO, examples of successful large-scale implementation are hard to find in published peer-reviewed health literature. In a small cross-sectional survey of 27 health centers in Addis Ababa, Ethiopia involving 506 presumptive TB cases, a substantial proportion (27.2%) of patients tested were found to have HIV but only 58.9% of participants were successfully tested for HIV [7]. In a similar study in Uganda, a higher uptake (85%) was reported, but the study was conducted in one large referral clinic and the generalizability of those results beyond similar clinics is questionable [8]. In a recent multi-clinic study in South India, designed to evaluate the prevalence of HIV among patients with presumptive TB, 85% of eligible patients were reported to have been tested for HIV [9]. PITC in those 3 studies was offered to patients with presumptive TB as part of the study and not as part of routine services. To help accelerate the scale-up of PITC for patients with presumptive TB in resource poor settings, examples of successful routine implementation in primary care settings as well as information on the burden of HIV among patients with presumptive TB are needed. Our study used data collected to monitor routine implementation of PITC for patients with presumptive TB in primary TB care clinics in the Democratic Republic of Congo. We aimed: (1) to assess uptake of HIV counseling and testing among patients with presumptive TB and (2) to quantify the burden of HIV among them.

## Methods

### Study design

This was a retrospective analysis of data collected to monitor and evaluate the implementation of PITC among patients seeking care in supported clinics with symptoms suggestive of TB.

### Setting and population

The DRC is a high TB and HIV burden country. Access to HIV testing remains extremely low. According to the recent national Demographic Health Survey, 68% of men and 63% of women have never been tested for HIV or do not know their HIV status [10]. In 2013, HIV status was known for only 44% of notified TB cases from DRC of whom 14% were HIV positive [3]. In 2010, the Schools of Public Health of the University of North Carolina at Chapel Hill (UNC) and of the University of Kinshasa (KSPH) with funding from the *President´s Emergency Plan For AIDS Relief (PEPFAR)* through the US Centers for Disease Control and Prevention (CDC), and in collaboration with the DRC’s National TB Program (PNLT) and the National AIDS Control Program (PNLS) started providing operational and logistical support to TB clinics in Kinshasa and in Kisangani, the capital of the Oriental Province (one of the provinces with the highest HIV prevalence). The objective of this collaboration was to support implementation of TB/HIV collaborative activities including routine implementation of PITC for all patients seeking care in the clinics with symptoms suggestive of TB. Before the UNC/KSPH program, routine HIV counseling and testing was only offered to patients with confirmed TB and the uptake was quite high [11, 12]. As part of the UNC/KSPH program, in each supported clinic, personnel involved in out-patient consultations were trained on TB symptoms screening, all patients with cough lasting more than two weeks or any other symptom suggestive of TB were routinely offered HIV testing and counseling. According to national guidelines, all patients diagnosed with HIV were offered cotrimoxazole prophylaxis onsite. HIV/TB patients were referred to HIV clinics for antiretroviral therapy (ART) after at least two weeks and within eight weeks of anti-TB treatment. HIV-infected patients with negative TB diagnosis were referred immediately to HIV clinics. TB diagnosis was based on the national algorithm (Programme Antituberculeux Integré IV) with at least one smear positive sample (for HIV-infected patients) and two positive samples for HIV-negative patients.

### Data collection and quality control

For monitoring purposes, all patients seeking care in the supported clinics with symptoms suggestive of TB were recorded using a different color of pen in the outpatient consultation registry by the consultation nurse/clinician. Before the patients were sent for further evaluation of their symptoms, they were counselled for HIV and a lab request was completed for HIV testing. Patients arriving at the laboratory for sputum testing who had not been counseled for HIV (without an HIV request) were referred to the TB nurse in the TB clinic for immediate counseling. Those who accepted to be tested for HIV and provided a blood sample were tested according to the national algorithm using three rapid tests and the results were recorded. At the end of each month, using information from the outpatients consultations, laboratory, and TB/HIV registers in each clinic, the number of patients with presumptive TB received in the clinic, the number of them counseled, the number tested for HIV, the number of those who tested positive as well as the number of TB patients and the number of HIV/TB patients recorded in each clinic were counted and recorded on the monitoring sheet. During the monthly visit by a member of UNC/KSPH technical team to the clinic, the team member verified the counts with the clinicians and laboratory technicians involved. The validated report was taken back to the central monitoring and evaluation team and entered into an electronic database, which was used for this analysis.

### Key outcomes, definitions, and analysis

PITC uptake was measured using two indicators: the proportion of presumptive TB patients counseled (calculated as the total number of patients recorded as counseled over the total number of patients with presumptive TB recorded in each clinic) and the proportion of counseled patients who received an HIV test. The prevalence of HIV was calculated as the proportion of patients tested who were diagnosed with HIV. An additional variable considered was the availability of onsite ART services (co-located in the same health facility as the TB clinics) or not. Chi square tests, Cochran Mantel Haenszel Odds Ratios (OR) and 95% Confidence intervals were used to compare the prevalence of key indicators by presence of onsite ART services. All analyses were stratified by calendar time, grouped in quarters. Analyses were performed using SAS 9.3 (Cary, NC) and all tests were performed at a 0.05 significance level. Use of the routinely collected data was approved by the University of North Carolina at Chapel Hill Institutional Review Board and the Kinshasa School of Public Health Ethical Committee.

## Results

### Uptake of routine PITC

In Kinshasa, 43,145 patients with presumptive TB were registered in 65 TB clinics between December 2011 and June 2013, including 33,602 (77.9%) patients from 30 clinics with and 9,543 (22.1%) from 35 clinics without onsite ART services. Of those, 27,797 (82.7%) and 8,466 (88.7%), respectively from sites with and without onsite ART services were counseled (Table 1). Of those counseled, 91.5% (n=25,430) and 95.3% (n=8,065) were tested for HIV in clinics with and without onsite ART services, respectively. Eligible patients in Kinshasa were less likely to be counseled and be tested for HIV in clinics with onsite ART services compared to patients in clinics without onsite ART services: OR 0.61 (95%CI 0.57, 0.65) and OR 0.71 (95%CI 0.64, 0.80) respectively In Kisangani, 6,687 patients with presumptive TB were registered in one of the 13 clinics between October 2011 and June 2013, including 3,832 (57.3%) in 7 clinics with and 2,855 (42.7%) in 6 clinics without onsite ART services (Table 2). Of those, 2,894 (75.5%) and 2,486 (87.1%) respectively in clinics with and without onsite ART were counseled and 2,859 (98.8%) and 2.486 (99.9%) were tested. Eligible patients in Kisangani were less likely to be counseled and be tested for HIV in clinics with onsite ART services compared to patients in clinics without onsite ART services: OR 0.51 (95% CI 0.44, 0.58) and 0.14 (95%CI 0.05, 0.41) respectively.

**Table 1:** Number of patients with presumptive TB (Suspects) and Number of TB cases recorded in 13 TB clinics in Kisangani and the proportion counseled, tested and found to be HIV positive by type of clinic attended (with and without onsite ART services) in Kinshasa, Democratic Republic of Congo, between October 2011 and June 2014.

Quarter	# of clinics a	Presump-tive TB cases b	Cases counseled cN (%)	Cases Tested dN (%)	HIV Positive eN (%)	TB cases f	Known HIV Positive gN (%)	TB cases tested hN (%)	Cases HIV Positive iN (%)
		**Onsite ART services^+^**							
Q4 2011	18	1778	455 (25.6)	441 (96.9)	80 (18.1)	515	27(5.2)	418 (85.7)	47 (11.2)
Q1 2012	23	5029	3793 (75.4)	3308 (87.2)	595 (18.0)	1660	60 (3.6)	1478 (92.4)	171 (11.6)
Q2 2012	23	4857	4329 (89.1)	3744 (86.5)	501 (13.4)	1827	47 (2.6)	1712 (96.2)	181 (10.6)
Q3 2012	28	5090	4660 (91.6)	4389 (94.2)	529 (12.1)	1600	31(1.8)	1512 (96.4)	176 (11.6)
Q4 2012	30	5122	4449 (86.9)	4140 (93.1)	464 (11.2)	1853	34 (1.9)	1749 (96.2)	162 (9.3)
Q1 2013	30	6286	5279 (84.0)	4897 (92.8)	634 (13.0)	1871	35 (2.7)	1722 (93.8)	210 (12.2)
Q2 2013	30	5440	4832 (88.8)	4511 (93.4)	624 (13.8)	1833	50 (2.7)	1733 (97.2)	209 (12.1)
**Total**		**33602**	**27797 (82.7)**	**25430 (91.5)**	**3427 (13.5)**	**11159**	**284 (2.5%)**	**10324 (94.9)**	**1156 (11.2)**
		**No onsite ART services^+^**							
Q4 2011	6	329	102 (31.0)	96 (94.1)	18 (18.8)	93	1 (1.1)	72 (78.3)	7 (9.7)
Q1 2012	9	882	681 (77.2)	593 (87.1)	89 (15.0)	394	5 (1.3)	344 (88.4)	32 (9.3)
Q2 2012	9	795	678 (85.3)	646 (95.3)	74 (11.5)	397	9 (2.3)	355 (91.5)	25 (7.0)
Q3 2012	26	1479	1382 (93.4)	1240 (89.7)	139 (11.2)	639	4 (0.6)	629 (99.1)	77 (12.2)
Q4 2012	35	1829	1686 (92.2)	1656 (98.2)	168 (10.1)	838	23 (2.7)	807 (99.0)	107 (13.3)
Q1 2013	35	2245	2045 (91.1)	2004 (98.0)	209 (10.4)	905	19 (2.1)	837 (94.5)	89 (10.6)
Q2 2013	35	1984	1892 (95.4)	1830 (96.7)	196 (10.7)	887	18 (2.0)	785 (90.3)	89 (11.3)
**Total**		**9543**	**8466 (88.7)**	**8065 (95.5)**	**893 (11.1)**	**4153**	**79 (1.9)**	**3829 (94.0)**	**426 (11.1)**

a Number of clinics contributing data

b Number of patients with presumptive TB registered.

c Number (N) and proportion (%) of patients with presumptive TB tested and counseled.

d Number (N) and proportion (%) of patients with presumptive TB counseled who were tested for HIV.

e Number (N) and proportion (%) of patients with presumptive TB who tested positive for HIV.

f Number of patients with confirmed TB diagnosis.

g Number (N) and proportion (%) of patients with confirmed TB diagnosis with known HIV positive status at time of TB diagnosis.

h Number (N) and proportion (%) of patients confirmed TB diagnosis tested for HIV.

i Number (N) and proportion (%) of patients with confirmed TB diagnosis who tested positive for HIV. Q4 2011 = fourth quarter of 2011.

*Onsite ART service = colocation in the same health facility as the TB clinic of an HIV services providing antiretroviral therapy (ART).

**Table 2:** Number of patients with presumptive TB (Suspects) and Number of TB cases recorded in 13 TB clinics in Kisangani and the proportion counseled, tested and found to be HIV positive by type of clinic attended (with and without onsite ART services) in Kisangani, Democratic Republic of Congo, between October 2011 and June 2014.

Quarter	# of clinics a	Presump-tive TB cases b	Cases counseled cN (%)	Cases Tested dN (%)	HIV Positive eN (%)	TB cases f	Known HIV Positive gN (%)	TB cases tested hN (%)	Cases HIV Positive iN (%)
		**Onsite ART services^+^**							
Q4 2011	5	507	364 (71.8)	362 (99.5)	27 (7.5)	184	14 (7.6)	159 (93.5)	17 (10.7)
Q1 2012	5	662	509 (76.9)	500 (98.2)	67 (13.4)	202	16 (7.9)	165 (88.7)	29 (17.6)
Q2 2012	5	542	371 (68.5)	365 (98.4)	52 (14.3)	208	21 (10.1)	155 (82.9)	20 (12.9)
Q3 2012	7	593	466 (78.6)	457 (98.1)	67 (14.7)	214	9 (7.3)	196 (95.6)	31 (15.8)
Q4 2012	7	521	399 (76.6)	391 (98.0)	64 (16.4)	205	15 (4.3)	189 (99.5)	44 (23.3)
Q1 2013	7	428	343 (80.1)	342 (99.7)	58 (17.0)	184	8 (2.5)	172 (97.7)	44 (25.6)
Q2 2013	7	579	442 (76.3)	442 (100.0)	47 (10.6)	243	6 (2.5)	237 (100.0)	42 (17.7)
**Total**	**7**	**3832**	**2894 (75.5)**	**2859 (98.8)**	**382 (13.4)**	**1440**	**89 (6.2)**	**1273 (94.2)**	**227 (17.8)**
		**No onsite ART services^+^**							
Q4 2011	1	650	475 (73.1)	475 (100.0)	30 (6.3)	68	10 (14.7)	48 (82.8)	12 (25.0)
Q1 2012	1	521	477 (91.6)	475 (99.6)	49 (10.3)	103	20 (19.4)	56 (67.5)	8 (14.3)
Q2 2012	1	479	414 (86.4)	414 (100.0)	34 (8.2)	119	12 (10.1)	74 (69.2)	12 (16.2)
Q3 2012	6	274	247 (90.2)	247 (100.0)	23 (9.3)	157	2 (2.5)	152 (98.1)	35 (23.0)
Q4 2012	6	321	321 (100.0)	321 (100.0)	31 (9.7)	201	5 (0.5)	196 (100.0)	30 (15.3)
Q1 2013	6	257	221 (86.0)	221 (100.0)	26 (11.8)	184	1 (1.9)	178 (97.3)	36 (20.2)
Q2 2013	6	353	331 (93.8)	331 (100.0)	44 (13.3)	257	5 (1.9)	252 (100.0)	36 (14.3)
**Total**	**7**	**2855**	**2486 (87.1)**	**2484 (99.9)**	**237 (9.5)**	**1089**	**55 (5.1)**	**956 (92.5)**	**169 (17.7)**

a Number of clinics contributing data

b Number of patients with presumptive TB registered.

c Number (N) and proportion (%) of patients with presumptive TB tested and counseled.

d Number (N) and proportion (%) of patients with presumptive TB counseled who were tested for HIV.

e Number (N) and proportion (%) of patients with presumptive TB who tested positive for HIV.

f Number of patients with confirmed TB diagnosis.

g Number (N) and proportion (%) of patients with confirmed TB diagnosis with known HIV positive status at time of TB diagnosis.

h Number (N) and proportion (%) of patients confirmed TB diagnosis tested for HIV.

i Number (N) and proportion (%) of patients with confirmed TB diagnosis who tested positive for HIV. Q4 2011 = fourth quarter of 2011.

*Onsite ART service = colocation in the same health facility as the TB clinic of an HIV services providing antiretroviral therapy (ART).

### Prevalence of HIV among patients with presumptive TB

In Kinshasa, of the 25,430 patients with presumptive TB in clinics with onsite ART tested for HIV, 3,427 (13.5%) tested positive. In clinics without onsite ART service, of the 8,065 tested, 893 (11.1%) tested positive for HIV (Table 1). Patients in clinics with onsite ART service were more likely to test HIV positive compared to those in clinics without: OR 1.21 (95%CI 1.12, 1.31). In Kisangani, of the 2,859 and 2,484 presumptive TB patients tested for HIV in clinics with and without onsite ART services respectively, 382 (13.4%) and 217 (9.5%), respectively, tested positive (Table 2). As in Kinshasa, patients from clinics with onsite ART service were statistically more likely to be HIV positive compared to those from clinics without: OR 1.53 (95%CI 1.28, 1.82).

### Prevalence of HIV among patients with diagnosed TB

In Kinshasa, during the evaluation period, 15,312 TB patients were registered in the participating clinics including 11,159 (72.9%) from clinics with onsite ART service and 4,153 (27.1%) from clinics without. Of those TB patients, 284 (2.5%) and 79 (1.9%), respectively in clinics with and without ART services were already known to be HIV positive. Of those with unknown HIV status at time of TB diagnosis, 10,324 (94.9%) and 3,829 (94.0%) were tested for HIV and 1,156 (11.2%) and 426 (11.1%), respectively, were found to be HIV positive. The prevalence of HIV was statistically similar in the two sets of clinics: OR 1.08 (95%CI 0.96, 1.22). In Kisangani, of the 2,529 TB patients reported from participating clinics including 1,440 (56.9%) and 1,089 (43.1%) from clinics with and without onsite ART services, 89 (6.2%) and 55 (5.1%), respectively were already known to be HIV positive. Of those with unknown HIV status at time of TB diagnosis, 1,273 (94.2%) and 956 (92.5%), respectively were tested for HIV and 227 (17.8%) and 169 (17.7%) were found to be HIV-infected. There was no statistical difference in the proportion of TB patients with HIV in clinics with and without onsite ART services: OR 1.05 (95%CI 0.84, 1.32).

## Discussion

This study aimed to assess the uptake of PITC for patients with presumptive TB in TB clinics and to quantify the burden of HIV among those patients. Our results showed that, PITC for patients with presumptive TB was relatively well accepted with over 85% of eligible patients counseled whether in Kinshasa or in Kisangani and over 90% of those counseled tested. In both cities, the proportions of patients counselled and tested were statistically lower in clinics with onsite ART services compared to clinics without. Inversely, in both cities, about 13.5% of patients tested in clinics with onsite ART services were found to be HIV positive, a prevalence that was statistically higher than the 11.1% and the 9.5% observed in clinic without onsite ART services, respectively in Kinshasa and Kisangani. On April 15, 2015, we search PubMed with the following terms (“provider-initiated” OR PITC OR PICT OR “routine testing” OR “opt-out”) AND (HIV OR AIDS) AND (TB OR Tuberculosis) and did not find any published paper reporting on large-scale implementation of PITC for presumptive TB in sub-Saharan Africa. The uptake of counselling and testing in our settings was higher than what was reported in small cross-sectional study from Ethiopia involving 506 patients (58%) [13] and comparable to the 85% that was observed in a relatively larger studies from India [14] and Uganda [8]. In all clinics involved in our study, HIV testing was done onsite and might explain the relative high uptake [12]. However, the proportion of patients with presumptive TB tested for HIV was lower than what was observed among confirmed TB cases. This was mostly the result of lower uptake of HIV counseling and testing in clinics with onsite ART services. The exact reason why the uptake was lower in clinics with onsite ART services is not known. However, as suggested by the higher prevalence of HIV among those who are tested in those clinics, it is possible that many patients registering in clinics with onsite ART services already knew their HIV status and were in the clinic simply to register for ART services. This hypothesis is supported by the higher proportion of TB patients in those clinics with known HIV status at time of TB diagnosis. Unfortunately, we did not collect information concerning previous HIV status for patients with presumptive TB.

Overall, whether in clinics with or without onsite ART services, the prevalence of HIV among patients with presumptive TB was quite similar in both Kinshasa and Kisangani and about 10 times the 1.2% prevalence in the general population suggesting that scaling-up PITC in primary care TB clinics in DRC is likely a cost-saving opportunity to speed-up identification of symptomatic HIV patients in a country where up to 68% of men and 63% of women never had an HIV test [10]. This study has a number of limitations: 1) only aggregated data were reported and available for this analysis making it difficult to link TB and HIV results. 2) As indicated above, a significantly lower proportion of patients in clinics with onsite ART services were counseled and tested. But because of the retrospective nature of the analysis, we are unable to verify if this was due to the fact that many of those patients knew their HIV status already. 3) We did not track patients diagnosed with HIV alone to assess their linkage to HIV care. But as part of routine services in all participating clinics, cotrimoxazole prophylaxis was initiated onsite and HIV infected patients were referred for HIV care and treatment. Results from tracking HIV/TB co-infected patients from those clinics reported elsewhere [15] show a high rate of successful linkages to ART services and timely initiation to ART for those eligible.

## Conclusion

In conclusion, implementation of PITC among patients with presumptive TB in primary care was well accepted and associated with high uptake of HIV testing among eligible patients. HIV testing among patients with presumptive TB in DRC yielded substantially higher proportions of HIV positive patients and could represent an effective way to rapidly identify symptomatic HIV-infected patients in need of treatment.

### What is known about this topic

- More than a decade after it was first recommended by the World Health organization (WHO), Provider initiated HIV testing and counseling (PITC) for patients with presumptive Tuberculosis (TB) is not widely implemented, particularly in the high TB and HIV burden countries in sub-Saharan Africa;
- Consequently, published literatures on the subject are limited to settings where PITC was offered to patients with presumptive TB as part of a study.

### What this study adds

- To the best of our knowledge, this is the first study reporting the outcome of a large-scale implementation of PITC for presumptive TB patients as part of routine care settings in primary health clinics in sub-Saharan Africa;
- Our results showed that: 1) large-scale implementation of PITC for patients with presumptive TB in primary care was well accepted and associated with high uptake of HIV testing among eligible patients and 2) yielded a proportions of HIV positive patients at least as high as PITCT for TB confirmed cases.
